# On Health Justice. Some Thoughts and Responses to Critics

**DOI:** 10.1111/bioe.12229

**Published:** 2015-12-21

**Authors:** Sridhar Venkatapuram

## Introduction

It is with enormous gratitude that I begin this response to the six articles evaluating, critiquing, and proposing improvements to different aspects of the argument I presented in Health Justice.^1^ I am extremely grateful to the guest editors of this collection of articles, namely Alena Buyx, Eszter Kollar, and Sebastian Laukötter. And, I must also thank the authors, the anonymous peer reviewers of these six articles, and the editors of this journal for their time, effort, and, essentially, support. Watching the making of this special issue has been a good reminder of how one's academic contributions are profoundly interwoven and interdependent with those of one's colleagues.

*Health Justice* was published first in the United Kingdom in late 2011, so it would be understandable to wonder why a series of articles responding to it are being published in early 2016. Journals will usually not review a book that is more than four years old. And most ‘author‐meets‐critics’ events and related publications happen right before or soon after the book's initial publication date. So the present special issue is, indeed, unusual. The book has for the most part received favourable reviews in different disciplinary journals, and has begun to motivate some doctoral dissertations as well as some policy and programmatic work. Interestingly, though, it has not appealed to American audiences as much as to academics and policymakers in Europe and developing countries. This is a puzzle that is true for the capabilities approach more generally. One of the editors of the *Journal of Human Development and Capabilities* recently pointed out that unlike most other academic journals this journal devoted to the capabilities approach sees more downloads of articles from outside the United States than from within.

The sociology of intellectual ideas has been a long standing interest of mine. Despite philosophers stating that they think abstractly and about ideas that will persevere for a long time, it is easy to see how social context shapes the scope and content of their ideas. For example, after reviewing a decade or more of issues of journals such as *Ethics* or *Philosophy and Public Affairs*, it is easy to trace a link between the published articles and the contemporaneous social context to which they were responding. One of the reasons I went to Cambridge to do my PhD was that finding academic support for developing a philosophical argument for a human right to health was easier there than at Harvard where I initially started graduate school. However, we also know that some ideas will not survive if they are not presented in a particular way, by certain people, or if the social context is not supportive. Do you know the story about the Dvorak keyboard? There are some social contexts where true ideas, facts, will simply not be believed. President Obama's birth certificate is one good example. On different occasions I have had to pause and think about the social context of advocating for a capabilitarian perspective to health justice. One event is worth noting. There is a journal called *Choice* which is important for the business of academic publishing as it reviews about 7000 academic books each year; it is a shopping list for university librarians. Seeing no review, we contacted them in late 2012 asking if they got a review copy. An editor replied that, no, they had not received the copy of the book. And, in any case, they only review books meant for American undergraduates. So I wrote back asking what about a book titled *Health Justice* or my name makes it seem that it is not meant for American undergraduates. They replied that it was too late for a review as the book was published in 2011.

The Munster symposium and the present collection of papers reflect certain features that I really value about working as an (American) academic in Europe. The participants are often well versed in the state‐of‐the‐art scholarly debates in the English‐speaking world. Yet they also provide alternative perspectives and propositions that one would not normally find in Anglo‐American environments. For me and other colleagues, discussions on health justice and global health justice must open up to include many more perspectives than what has largely been dominated so far by Anglo‐American philosophers. This is also becoming recognized in global justice philosophy more broadly. Just within Europe there are distinctly different ethical approaches and arguments regarding health and social justice among philosophers in the UK, Netherlands, Germany, Austria, and Spain. We have to hear these voices. The fact that the present collection of articles contains perspectives of individuals from the far corners of the world is largely due to the exceptional skills of the guest editors. They have trained and worked in various countries including the United States, and are now based in Germany. They are producing scholarly work that is engaged with the state of the art of debates in the fields of bioethics and political philosophy, is of the highest quality, and importantly, they speak coherently and with relevance to audiences across many countries. They do not slip easily or carelessly into universal language. So it is both a great pleasure and a privilege to be associated with this new generation of international philosophers.

## Critiques

At the outset, I must acknowledge how much I have benefitted from reading and reflecting on the six articles by the eight contributors to the present special issue. Indeed, all of the authors present substantial criticisms of various aspects of the argument presented in *Health Justice*. However, most of them also generously suggest ways to amend the argument to address their criticisms. Importantly, rather than simply criticizing, each of the articles teaches in its own way on various topics in relation to their criticism. I cannot think of better kinds of critics. The contributors would agree with me, I believe, that there could be an endless supply of both major and minor criticisms of the argument in *Health Justice*. What can be frustrating is when criticisms are based on misunderstandings, wilful misrepresentations or are just misdirected. This is clearly not the case of the present authors. I begin first with motivation for the argument, then a brief summary of the argument, before addressing the criticisms. I am unable to address every criticism made by the contributors, nor do I think I am expected to do that. I have tried to be diligent about addressing at least the major criticisms of each contribution.

In *Health Justice*, I strive to make a moral right to health a coherent and viable concept by extending the capabilities approach (CA) to the health domain. Put simply, the aim is to bring rights, health, and capabilities together in a way that could be practicable as well as philosophically coherent. Some scholars represent the CA as a partial theory of social justice and others see it is an approach to social justice. It is also presented as something even more abstract than a theory of justice; that it is an intellectual discipline or ethical theory.^2^ In any case, since the basic ideas of the CA were articulated by Sen in 1981,^3^ it has developed to the point where it is generally accepted that it is a plausible candidate theory for grounding basic moral rights for human beings as well as for the human rights articulated in international law.^4^ Sen had from the start linked capabilities to rights.^5^ I have been interested in linking the CA and human rights since the early 1990s. My initial exposure to the CA was in 1989 when I heard Amartya Sen deliver a lecture on famines. I then used his entitlement analysis of famines, and Martha Nussbaum's arguments regarding basic human capabilities, to write a paper on women and HIV/AIDS for her course on feminist philosophy in 1994. Informed by Sen's entitlement analysis and Nussbaum's arguments for moral claims to central human capabilities, my undergraduate dissertation argued that addressing the global HIV/AIDS epidemic will require integrating and promoting both civil and political rights as well as economic and social rights. This was motivated by an exchange I had while working at Human Rights Watch where it was explained to me by the Director that the right to health and all the economic and social rights were misconceived. This interest in the link between health and the philosophy of human rights continued in graduate school where I worked with the UN Special Rapporteur on the Human Right to Development to develop its conceptual framework, and wrote a dissertation on health rights in international law and philosophy in 2000.

Starting in the late 1990s, both Sen and Nussbaum had begun to articulate the prospect that the CA could provide the philosophical grounding to human rights.^6^ That is, through a process of global public deliberation, certain basic human capabilities or freedoms can be identified as valuable and given the status of moral entitlements or human rights. Encouraged by the plausibility of human rights to capabilities, in my doctoral dissertation, I set out to construct a moral/human right to the capability to be healthy. This would then be the basis of *Health Justice*. To give this argument coherence and stability across different relevant disciplines, I formulated health as a capability, specifically a ‘meta‐capability’ of capabilities. Second, I showed how this capability to be healthy fits in with the latest insights in social epidemiology, with supporting references to economic modelling of capabilities such as in the case of famines. Third, I situated the argument for a CH within the capabilities approach, and showed how a capability can be formulated as a ‘cluster right’. Fourth, I defended it against the dominant approaches namely the Rawlsian approach developed by Norman Daniels, and utilitiarianism, such as in cost‐efficiency analysis using QALYs and DALYs. And lastly, I discussed the problems of group capabilities and global justice, and strategies for how the CH could address them. As I state in the book, the argument presented is the first instalment. There are many aspects to be developed, particularly regarding the implementation or application of the concept. The fundamental aim of the book was to set out the basic framework for a moral right to the capability to be healthy, and its centrality to social and global justice. The contributions of the current special issue will be important influences on the next instalment of the argument.

Four of six critics including Richardson, Schramme, Tengland and Selgelid target their criticisms on the formulation of health as meta‐capability. I will first address the conception of health critiques and then address the other two contributions. In brief, Richardson argues that I should pull back my conceptualization of health from the ability to achieve or exercise ten central capabilities to just the non‐voluntary biological processes that are the bases of the ten basic capabilities. Schramme argues that condensing the ten basic capabilities into one meta‐capability to be healthy does not solve the CA's metric or threshold problems. He also does not see the justification of health conceptualized as the ability to achieve ten basic capabilities. He advises that it would be better to go with the disease conception of health as it is easily justifiable, and provides clear methods to establish justice claims and their limits. Tengland favours a conception of health as simply having some basic first order abilities, possibly akin to Richardson's suggestion. Selgelid, among a long list of criticisms, argues that health as capability is greatly at odds with usual conventions, and that a claim to the capability to achieve the capability to X or Y seems to be verging on the nonsensical, exhibiting a form capabilities fetishism. Why not just a claim to X and Y?

In the CA, the basic building blocks are the individual, and her capabilities and functionings. A person's capability to do or be something reflects the practical possibility of her doing or being something. Such a capability or practical possibility is made up of the combined interaction of her individual endowments and skills and her external environment. A person's capability is clearly not just about her personal or internal traits or skills; a capability is an assessment of person's ability to be and do something situated in her environment. Functionings is a term to represent the actual outcomes or achievements of the capabilities. For a variety of reasons, the quality of life of an individual, (how well their life is going or their wellbeing) is argued to be best conceptualized or evaluated in the space of her capabilities. This space of capabilities is a more coherent space to evaluate wellbeing, it is argued, than a person's actual beings and doings, resource holdings such as income, or happiness, liberties, basic needs, opportunities for welfare, and so forth. Furthermore, CA theorists argue that the target of the moral concern about inequality is also best directed towards the space of capabilities.

Richardson and Tengland do not have any issue with these aspects of the CA. Schramme and Selgelid do have concerns. While Schramme appreciates the discussions about the merits of the capability space over other targets of moral concern, he does not see how the CA framework allows us to assess whether one individual who is deprived in one capability is better or worse off than another individual deprived in a different capability space. Selgelid raises the point that ‘capabilities largely matter because they promote freedoms and enable achievement of wellbeing. Well‐being itself, however, is ultimately important’ he says. As these points regard the fundamentals of the CA, I defer to the large body of literature by Sen, Nussbaum and other colleagues.^7^ But briefly, to respond to Schramme's point, Sen does not offer an easy method for assessing or comparing the capabilities of two individuals. If capabilities are to be understood as freedoms, freedoms of different kinds are also different social values. Thus, the question being raised is about the commensurability of different values. Furthermore, Sen has argued that the measurement of capability needs to also be supplemented by contextual analysis, and sensitive to process, non‐domination, non‐discrimination, and so forth. As a result, Sen and other CA theories readily accept that there will not always be full social agreement on which individual is better off than another, or how to rank different scenarios of wellbeing. However, Sen suggests that great progress can be made through efforts at achieving ‘partial orderings’; and it is why public deliberation and impartial reasoning have such significant roles in his approach. In Nussbaum's partial theory, two individuals not having sufficient capabilities in different domains are incomparable; they are both deprived in distinctly different ways. A comparison of two capability domains in order to make trade‐offs is not allowed.

In regard to Selgelid, I don't think he misunderstands here the important value the CA places on self‐determination, or respecting the choice of individuals to plan and pursue their life plans. In the cases of children, and other dependent adults, the CA does aim for achievements rather than just ensuring capabilities. But I think Selgelid is making a different kind of point. He writes that wellbeing can be seen as other things than just positive mental states; it can include a range of plural goods such as autonomy, happiness, satisfaction and other objective lists of goods. Thus, a list of ‘objective’ capabilities is too removed from all these kinds of possible plural goods of well‐being. Furthermore, he disagrees that the target of social policy as being wellbeing achievements or functionings always violates the personal choice principle. While I acknowledge these points, they are too general and require more elaboration to give a good response. Otherwise, they seem to require the rehearsing of the basic theory of the CA.

A core contribution of *Health Justice* is its conceptualization of health as a capability. Sen and Nussbaum as well as other CA theorists often referred to health in the extant literature at that time as being an important or central capability, but the underlying conception of health was unclear. There were references to health as being important for achieving basic capabilities, inequities in capabilities to achieve health, health capability paradigm in healthcare, and so forth. I saw many ways of conceptualizing health as a capability. Perhaps the least philosophical was derived from the practical, public health work of preventing HIV infections and the literature related to efforts to improve the sexual and reproductive health of girls and women in developing countries. The central idea from effective efforts and health services research was that for an individual to protect themselves from a fatal or debilitating infection requires the effective combination of individual knowledge, skills, and agency as well as an environment with supportive social and physical conditions. This line of reasoning was commensurate with Sen's analysis of the acute and endemic starvation (famines). The ability of a person to protect themselves from a fatal or debilitating condition was akin to the ability of a person to be adequately nourished. Borrowing from Sen even more, it is not the availability of commodities in the locality, or the person's happiness that is a good reflection of their health but her abilities to protect, maintain, and promote her beings and doings. Such an analysis also makes clear how the availability of healthcare is an important component, but cannot be the totality of the concern regarding health; we need to focus on the abilities of each individual in light of her internal endowments and skills and external environment.

The recognition of the importance of capability versus commodities or mental welfare, however, does not address a related but conceptually separate issue. In the case of famines, when we recognize that the capability to be well nourished is a distinct and important space to monitor in contrast to the space of actual nourishment achievements (functionings), we are not also saying that the capability to become well‐nourished is, in fact, being well nourished. The abilities to become well nourished are distinct from actually being well nourished. So it seems incorrect to say that because we should be concerned about the abilities to be/become healthy, the abilities or capabilities themselves constitute health, or being healthy. The means to achieve health, or the determinants, are being conflated with the constitutive components of health. Schramme, Tengland, and Selgelid all point to this error.

However, when we look towards popular or common language notions of health, there is often a sense of being healthy as being able to do things, and without any pain or physical and mental constraint. In fact, it is not just that being healthy is being able to do something, being healthy is also about the having the practical possibilities of doing a wide array of things. The welfare or holistic theories of health start from this popular intuition, and develop a conception of health which is centred on an individual being able to be and do things in their environment. Lennart Nordenfelt argues that health should be understood as the assessment of the abilities to achieve vital goals. Or more specifically, that health is an assessment of the second order abilities to achieve vital goals in standard circumstances. The second order ability is important here, as Nordefelt argues, correctly in my view, because being unable to achieve one's vital goals has to mean that one is unable to achieve a vital goal in standard circumstances as well as one is unable to learn or recover the ability to achieve one's vitals goals. Learning or recovering the ability to achieve vital goals put you on your way to being healthy. Conversely, to be healthy is more precisely having the second‐order ability to achieve vital goals; to be able to develop the ability. Despite it sounding odd, the capability to be capable of doing something is not so foreign to the CA. However, Tengland rejects the need for this second‐order ability, and prefers a conception of health that is simply having some basic physical and mental abilities. Simply put, a person who is undernourished is unhealthy. It is hugely counterintuitive, according to Tengland, to say that they are unhealthy only when they also lack the ability to learn or recover their ability to be nourished. I would argue that Tengland is taking the conceptual corollary necessary to clearly assert that someone is not able to achieve a vital goal and thus, not healthy, and over‐exaggerating it as a conceptual weakness of the idea of health as being capable to achieve a cluster of basic capabilities. I defer here to Nordenfelt, who does a sufficient job explaining second‐order ability.^8^

The language of capabilities and functionings was initially used by Sen and Nussbaum to put across some basic concepts. They were concepts that Nussbaum borrowed from Aristotle. However, most CA theorists and advocates, I would argue, recognize that one cannot describe flesh and blood human beings only in terms of a simple set of capabilities and functionings. Even Nussbaum, who argues that a person with a life with minimum human dignity has at least certain ten basic capabilities, recognizes that the ten capabilities are conceptually distinct but are inter‐dependent. And that as people go through life, their capabilities lead to functionings, which can then lead to more capabilities, and so forth. Jonathan Wolff and Avner De‐Shalitt identified what they call ‘corrosive disadvantage’ and ‘fertile functionings’.^9^ That is, the lack of certain capabilities can be grossly damaging to other capabilities and functionings, while some functionings can be hugely beneficial to engendering other capabilities and functionings. Capabilities can be affected by or dependent on other capabilities and functionings. So rather than being non‐sensical and fetishistic, as Selgelid claims, there is plausibility in the understanding that being capable to achieve X can be a constitutive component of being capable of achieving Y. Or, having the capability to X means having the capability to develop the capability Y.

One way I modify Nordenfelt's concept of health as the ability to achieve vital goals is that I fill in the vital goals with Nussbaum's ten central human capabilities. As she states, her list is not exhaustive, but the ten capability domains are a fixed minimum. Because I want to define health as the ability to achieve ten vital goals/basic capabilities, I specify Nussbaum's second capability which refers to bodily health as relating to disease and impairment. This means that being able to prevent, mitigate or recover from disease and impairment is an important component of health but only one of a cluster of components. Richardson, Schramme, and Selgelid press that point that I have too easily abandoned the concept of disease, especially the Boorsean notion. But I have not. Boorse's conception of disease can plausibly still live in the second capability. At this point, health is conceptualized as a cluster of ten basic human capabilities that are constantly developing, interdependent, and iterative. And applying the second‐order ability component of Nordenfelt's definition gives us the ability to achieve a cluster of basic capabilities; a meta‐capability.

Some critics point out that while I may have used Nordenfelt's framework for a definition of health, I have somehow missed doing two things. Selgelid states that I have said very little about the relationship between each of the basic capabilities and health. And second, I have not justified a conception of health as being a cluster of basic capabilities. I have to accept these two points. In the search for a usable conception of health in capability theory, Nordenfelt's framework seemed ready made and insightful. Once I amended his theory of health from the ability to achieve vital goals to achieve basic capabilities, I assumed the job was done. I suggested that if there were objections to this particular list of basic capabilities, there are possibilities of producing a different set, perhaps using different methods from those Nussbaum used. So, given that I defined health as being constituted of ten basic capabilities, I did not see how describing each capability's link to health was going to be more helpful. Regarding justification, the defining of health in term's of Nussbaum's ten central human capabilities would provide both the ethical reasoning for the value of each capability as well as the justification she offers for the moral claims of the central human capabilities. Furthermore, given how notoriously difficult previous attempts have been to separate health from wellbeing, the minimal conception of wellbeing developed by Nussbaum could also help to contain health from expanding into total wellbeing. From the start, I also rely on Sen's conceptions of capabilities as basic freedoms and his methods of justification. The basic capabilities that constitute health were grounded in both dignity and freedoms. So Schramme's assertion that I have hidden or elided the need to justify the value of health conceived as ten basic capabilities is unfair. I am open to criticisms about health as the ability to achieve basic capabilities, but for justification of the value of the basic capabilities or freedoms, I rely on Sen and Nussbaum.

Richardson presents the critique that ‘health cannot be *the* metacapability of having central human capabilities, nor can it be *a* metacapability of having the central human capabilities.’ If I understand correctly, his worry starts with the recognition of health as being distinct from other capabilities and important goods such as income, and the ‘the criss‐crossing web of causal dependencies among health, education, income, and wealth and each of the ten central human capabilities’. Another concern follows from the understanding that having a capability to X is meant to imply the practical possibility of achieving X. Then, according to my definition of health, a capability to be healthy implies that individuals have the practical possibility to achieve all ten central human capabilities. Given that many societies in the world do not or cannot provide the social environments to ensure the practical possibility of achieving ten central capabilities, then in many countries no one is healthy. It seems counter‐intuitive to Richardson to accept that the extent of how health a person is varies directly with features of their country's constitutional regime, robustness of job market, and reach of educational system. The social determinants of health become just too expansive. The solution that Richardson proposes is to define health relating to the non‐voluntary, bodily substrate or bodily processes related to basic capabilities including respiration, immune response, blood clotting and so forth. While they are non‐voluntary, they would fall under Sen's understanding of ‘effective freedom’. That is, the individual would have chosen such a capability or functioning had she been able to choose. So, Richardson suggests the amendment which would result in the definition that ‘health is the non‐voluntary, bodily aspect of the metacapability for the central human capabilities.

While Richardson's insightful and generous evaluation is illuminating, I will hold off from readily accepting the amendment. I am not troubled by the idea of the health capability of individuals is determined by national and even global factors. For example, the lack of a functioning education system precludes individuals from becoming literate, and this reduces their wellbeing. If health was understood as a minimal conception of wellbeing, and it includes a literacy capability, then than the lack of a functioning educational system reduces or constrains that person's health. The analysis of the broad social factors on the beings and doings of individuals, their health and wellbeing, is the bread and butter work of social epidemiologists and macroeconomics. The related point is that it seems too counter‐intuitive to accept that based on the health capability idea, that the majority of people in a country are not healthy. This worry may be tempered by a closer look at Nussbaum's list of basic capabilities and her sufficiency thresholds. They are really not as demanding as some people imagine. She argues for social conditions that provide basic literacy and numeracy, not formal education up to a certain standard. At the same time, the real power of this argument for a health metacapability is to reveal the true extent of the many human beings who are below a minimal level of wellbeing. Just as our understanding of human deprivation was transformed when we moved from measuring premature mortality to include disability, we will see yet more when move to measuring health capability. The multidimensional poverty index is a move in such a direction.^10^

## Vulnerability and Individual Autonomy

Christine Straehle's contribution is both surprisingly original and welcome. I learned a great deal about a subject that I knew little about, namely liberal individual vulnerability. Her assertion is that health should not be considered as a meta‐capability or pressed as a strong or central claim of social justice. Using various examples of vulnerability, she argues that there are certain background conditions of vulnerability that affect the health‐enabling decisions of individuals, where health capability social interventions cannot reach, therefore, revealing the concept's limits. The argument is even stronger as she argues that these background conditions of vulnerability may provide a case against the strong claim that health capability is central to social justice; in such conditions it may be more important to support other capabilities, not health capability. While I am intrigued with the final example, and acknowledge the force of her conclusion, I am confused by the final section. The penultimate paragraph juxtaposes my argument for health as meta‐capability next to Nussbaum's list of capabilities. Straehle then states that the case may require that it would be better to promote Nussbaum's capability to control one's environment rather the health metacapability. This raises a worry that Straehle has missed the important part of my argument where health metacapability is defined as the capability to achieve Nussbaum's ten central capabilities. This confusion seems to undermine her move to build the case against health capability being central to social justice. However, it would be still be interesting to consider how a health metacapability, properly understood as a capability to achieve a cluster of ten basic capabilities, would deal with the final scenario. I also have concerns that my health capability argument may have been conflated with other capabilities the work of scholars who talk more about health agency. Lastly, Sen has argued that aside from the capability space, it is also important to examine the context, process concerns, ensuring non‐domination, non‐discrimination and so forth. It seems to me that in addition to these dimensions, vulnerability is also something to be examined.

## Global Health Justice

The contribution of Kollar and colleagues focuses on the global health duty that arises from the argument for every human being's right to the capability to be healthy, wherever we find them. They write that in its current form, the argument gives rise to humanitarian duties rather than demands of justice. They then generously explain how a more explicit focus on the social determinants of health would make a stronger candidate for producing duties of justice. The present weaknesses, according to Kollar and colleagues are that Nussbaum's list of central capabilities has many substantial criticisms against it, and that its grounding in the concept of equal human dignity gives rise to humanitarian duties not demands of global health justice. Furthermore, the CA more broadly lacks some crucial elements of a theory of justice such as an account of injustice and obligation. The weakness specific to my particular argument for health capability, they claim, is that I do not attach any normative significance to the causes of the deprivations and failures of health capability. We do not know whether a capability deprivation was caused by some agent or if it merely happened. The former would raise a justice duty while the latter would be a humanitarian duty.

Let me first address the issue about relying on Nussbaum's arguments for basic human capabilities. I am assuming that they have concluded that the list of basic capabilities grounded in equal human dignity is not sufficiently justifiable. And even if it was, that it gives rise to humanitarian duties to protect the health capability of foreigners, rather than demands of justice. Selgelid is much less charitable about relying on the concept of dignity. He writes that it is unfortunate that I tie my concept of health to dignity as many bioethicists now consider it to be meaningless. Furthermore, he points out that Nussbaum's reasoning about dignity has been examined closely and was found to reflect the kind of confusion bioethicists worry about. This scrutiny and criticism of Nussbaum's list or grounding in dignity is not unexpected. The health capability argument relies on both Nussbaum's dignity and Sen's freedoms. As my focus was largely on developing the concept of health as capability, I did not try to find alternative ethical grounding for capabilities. While I appreciate the worry about dignity, I am not willing to give it up just yet, even if all bioethicists have abandoned it. At the same time, Sen's grounding of capabilities in the fundamental value of freedom, and his method of justification, make it even more difficult to derive global health duties. So, I do accept the criticism that the fundamental justifications for basic capabilities need to be examined more closely.

Finally, while focused on the lack of normative attribution of the causes, and duties of justice, I think Kollar and her colleagues have missed out on a lot more. I have argued that the moral concern for health is multi‐dimensional – there are moral dimensions to the causes, consequences, levels, distributions patterns, experience, and so forth. And I did, indeed, discuss who has what kind of duties in relation to the multiple‐dimensions of the capability to be healthy (156‐57). In the global context, I suggested that Thomas Pogge's arguments for negative, positive, and intermediate duties have a long reach. However, I am not sure how much the relational view will cover all these various dimensions. They are indeed correct that I did not spend enough time discussing the normative role of the social determinants of health both domestically and globally. Their contribution is very illuminating, and helpful in motivating me to focus more energy on the global health justice and duties aspect of social determinants of health. I believe that it will also motivate a number of other individuals, including some colleagues who are philosophers of causation and explanation.

## Footnotes

1

S. Venkatapuram . Health Justice. An Argument from the Capabilities Approach. Cambridge: Polity Press; 2011.

2

I. Robeyns . The Capability Approach [Online]. Stanford: Stanford University; 2011 Available: http://plato.stanford.edu/archives/sum2011/entries/capability-approach/ [cited 2011 April 19].

3

A. Sen . Rights and Agency. Philos Public Aff 1981; 2: 3–39.

4

A. Sen . Equality of What? Choice, Welfare, and Measurement. 1st MIT Press ed. Cambridge MA: MIT Press; 1982; A. Sen . The Idea of Justice. London: Allen Lane; 2009; M.C. Nussbaum . Creating capabilities : the human development approach, Cambridge MA/ London: Belknap; 2011.

5

A. Sen . Rights and Capabilities Resources, Values and Development. Cambridge: Harvard University Press; 1999; A. Sen . Rights as Goals. Annual Conference for Legal and Social Philosophy. University College: London; 1984; A. Sen . Rights and Agency. Philos Public Aff 1981; 2: 3–39.

6

A. Sen . Elements of a Theory of Human Rights. Philos Public Aff 2004; 32: 315–355; M.C. Nussbaum . Capabilities and Human Rights. Fordham Law Review 1997; 66: 273–300.

7
Sen 2009, *op. cit* note 5; Nussbaum 2011, *op. cit* note 4; Robeyns 2011, *op. cit*. note 2.

8

L. Nordenfelt , G. Khushf , K.W.M. Fulford . Health, Science, and Ordinary Language, Amsterdam: Rodopi; 2001.

9

J. Wolff , A. De‐Shalit . Disadvantage, Oxford: Oxford University Press; 2007.

10

S. Alkire , M.E. Santos . Acute Multidimensional Poverty: A New Index for Developing Countries OPHI Working Papers. Oxford: Oxford Poverty & Human Development Initiative; 2010.

